# The impact of UEFA Euro 2020 football championship on Takotsubo Syndrome: Results of a multicenter national registry

**DOI:** 10.3389/fcvm.2022.951882

**Published:** 2022-09-28

**Authors:** Alberto Polimeni, Carmen Spaccarotella, Jessica Ielapi, Giovanni Esposito, Amelia Ravera, Eugenio Martuscelli, Vincenzo Ciconte, Maurizio Menichelli, Ferdinando Varbella, Massimo Imazio, Alessandro Navazio, Gianfranco Sinagra, Rainer Oberhollenzer, Gerolamo Sibilio, Luisa Cacciavillani, Luigi Meloni, Marcello Dominici, Fabrizio Tomai, Francesco Amico, Marco Corda, Giuseppe Musumeci, Alessandro Lupi, Luigi Zezza, Raffaele De Caterina, Carlo Cernetti, Marco Metra, Lidia Rossi, Paolo Calabrò, Adriano Murrone, Massimo Volpe, Pasquale Caldarola, Stefano Carugo, Bernardo Cortese, Renato Valenti, Giuseppe Boriani, Francesco Fedele, Giorgio Ventura, Maria Teresa Manes, Angela Rita Colavita, Mauro Feola, Francesco Versaci, Pasquale Assennato, Giuseppe Arena, Roberto Ceravolo, Vincenzo Amodeo, Gianfranco Tortorici, Daniele Nassiacos, Roberto Antonicelli, Nicolino Esposito, Stefano Favale, Giovanni Licciardello, Luigi Tedesco, Ciro Indolfi

**Affiliations:** ^1^Division of Cardiology and Center for Cardiovascular Research, University Magna Graecia, Catanzaro, Italy; ^2^Division of Cardiology, Department of Advanced Biomedical Science, Federico II University, Naples, Italy; ^3^San Giovanni di Dio e Ruggi d'Aragona Hospital, Salerno, Italy; ^4^Dipartimento di Medicina Interna, Divisione Cardiologia, Università degli Studi di Roma Tor Vergata, Rome, Italy; ^5^Azienda Ospedaliera Pugliese-Ciaccio, Catanzaro, Italy; ^6^Ospedale Fabrizio Spaziani, Frosinone, Italy; ^7^Azienda Ospedaliero-Universitaria San Luigi Gonzaga, Orbassano, Italy; ^8^Cardiology, Cardiothoracic Department, University Hospital “Santa Maria della Misericordia”, ASUFC, Udine, Italy; ^9^IRCCS Azienda Unità Sanitaria Locale di Reggio Emilia, Reggio Emilia, Italy; ^10^Dipartimento Cardiotoracovascolare, Università degli Studi di Trieste, Trieste, Italy; ^11^San Maurizio Regional Hospital, Bolzano, Italy; ^12^Santa Maria delle Grazie Hospital, Pozzuoli, Italy; ^13^Dipartimento Cardiotoracovascolare, Università degli Studi di Padova, Padua, Italy; ^14^Dipartimento di Cardiologia, Università degli Studi di Cagliari, Cagliari, Italy; ^15^Azienda Ospedaliera Santa Maria, Terni, Italy; ^16^European Hospital, Rome, Italy; ^17^Cardiologia UTIC Emodinamica, Catania, Italy; ^18^SC Cardiologia UTIC, ARNAS “G. Brotzu”, Cagliari, Italy; ^19^SC Cardiologia, AO Ordine Mauriziano, Turin, Italy; ^20^Dipartimento di Cardiologia, Università degli Studi dell'Insubria, Varese, Italy; ^21^Card. G. Panico” Hospital, Tricase, Italy; ^22^Divisione di Cardiologia, Università di Pisa, Pisa, Italy; ^23^Azienda ULSS 2 Marca Trevigiana, Treviso, Italy; ^24^Divisione di Cardiologia, Università degli Studi di Brescia, Brescia, Italy; ^25^Azienda Ospedaliero-Universitaria Maggiore della Carità, Novara, Italy; ^26^Università degli Studi della Campania Luigi Vanvitelli, AORN Sant'Anna e San Sebastiano Caserta, Caserta, Italy; ^27^Ospedali di Città di Castello e Gubbio - Gualdo Tadino Azienda USL Umbria 1, Perugia, Italy; ^28^University of Rome Sapienza and Sant'Andrea Hospital, Rome, Italy; ^29^Ospedale San Paolo, Bari, Italy; ^30^Cardiology Unit, Internal Medicine Department, Fondazione IRCCS Ca' Granda Ospedale Maggiore Policlinico, University of Milan, Milan, Italy; ^31^Cardiac Department Clinica Polispecialistica San Carlo, Milan, Italy; ^32^Fondazione Ricerca e Innovazione Cardiovascolare, Milan, Italy; ^33^Azienda Ospedaliera Careggi, Florence, Italy; ^34^Divisione di Cardiologia, Dipartimento di Scienze Biomediche, Metaboliche e Neuroscienze, Università degli Studi di Modena e Reggio Emilia, Policlinico di Modena, Modena, Italy; ^35^Sapienza Università di Roma, Rome, Italy; ^36^Istituto Ninetta Rosano - Casa di Cura Polispecialistica Tricarico, Belvedere Marittimo, Italy; ^37^U.O.C. Cardiologia, Cosenza, Italy; ^38^U.O.C. Cardiologia/UTIC PO Cardarelli, Campobasso, Italy; ^39^Regina Montis Regalis Hospital, Mondovi, Italy; ^40^Department of Cardiology, Santa Maria Goretti Hospital, Latina, Italy; ^41^Dipartimento di Medicina, University of Rome Tor Vergata, Rome, Italy; ^42^Azienda Ospedaliera Universitaria Policlinico Paolo Giaccone, Palermo, Italy; ^43^Ospedale Apuane, Massa, Italy; ^44^ASP Catanzaro, Lamezia Terme, Italy; ^45^Ospedale Santa Maria degli Ungheresi, Polistena, Italy; ^46^Ospedale di Bentivoglio, Bentivoglio, Italy; ^47^ASST Valle Olona, Busto Arsizio, Italy; ^48^U.O.C. di Cardiologia-UTIC, INRCA-IRCCS, Ancona, Italy; ^49^Divisione di Cardiologia, Ospedale Evangelico Betania, Naples, Italy; ^50^Università degli Studi di Bari Aldo Moro, Bari, Italy; ^51^Augusta Hospital, Augusta, Italy; ^52^Presidio Ospedaliero S. Maria della Speranza, Battipaglia, Italy; ^53^Mediterranea Cardiocentro, Naples, Italy

**Keywords:** cardiomyopathies, ACS, angina pectoris, Takotsubo, cardiovascular disease

## Abstract

**Objectives:**

The UEFA 2020 European Football Championship held in multiple cities across Europe from June 11 to July 11, 2021, was won by Italy, providing an opportunity to examine the relationship between emotional stress and the incidence of acute cardiovascular events (ACE).

**Methods and results:**

Cardiovascular hospitalizations in the Cardiac Care Units of 49 hospital networks in Italy were assessed by emergency physicians during the UEFA Euro 2020 Football Championship. We compared the events that occurred during matches involving Italy with events that occurred during the remaining days of the championship as the control period. ACE was assessed in 1,235 patients. ACE during the UEFA Euro 2020 Football Championship semifinal and final, the most stressful matches ended with penalties and victory of the Italian team, were assessed. A significant increase in the incidence of Takotsubo Syndrome (TTS) by a factor of 11.41 (1.6–495.1, *P* < 0.003), as compared with the control period, was demonstrated during the semifinal and final, whereas no differences were found in the incidence of ACS [IRR 0.93(0.74–1.18), *P* = 0.57]. No differences in the incidence of ACS [IRR 0.98 (0.87–1.11; *P* = 0.80)] or TTS [IRR 1.66(0.80–3.4), *P* = 0.14] were found in the entire period including all matches of the UEFA Euro 2020 compared to the control period.

**Conclusions:**

The data of this national registry demonstrated an association between the semifinal and final of UEFA Euro 2020 and TTS suggesting that it can be triggered by also positive emotions such as the victory in the European Football Championship finals.

## Introduction

Football is a highly popular sport in many countries worldwide. Few updated data exist on the cardiac hospitalizations, acute coronary syndromes (ACS), and Takotsubo Syndromes (TTS) during the football championships, especially in the contemporary era, debating the association of acute cardiovascular events (ACE) with watching football matches.

The UEFA Euro 2020 Football Championship, commonly referred to as UEFA Euro 2020, was the 16th UEFA European Championship. The tournament was won by Italy, who in the final match beat England on penalties 3-2, after the 1-1 in extra time. In Italy, the emotional participation of the general population in the European football championship is robust and this year was amplified because Italy entered the semifinals and the final.

The relationship between spectators' emotions and ACE during sporting events has been reported with conflictual results (1–5).

Accordingly, the present study aimed to assess the effect of the UEFA Euro 2020 Football Championship on hospitalizations for ACE in Italy.

## Methods

ACE were assessed in an Italian Registry that included 1,235 patients admitted to 49 cardiac care units across all of Italy. The distribution of patients enrolled was geographically balanced (North Italy 34%, Center 33%, South 32%). All patients' data were collected and reported into an electronic database.

The demographic and clinical data of the subjects along with the type of cardiovascular diseases were evaluated. Physicians made the clinical diagnosis of ACS or TTS based on the symptoms, the level of the markers of myocardial necrosis, EKG, Echocardiogram, and coronary angiography according to the current guidelines.

Italy's matches during the UEFA Euro 2020 Football Championship were 7 in total, and they were held on: 11 June (Italy vs. Turkey), 16 June (Italy vs. Switzerland), 20 June (Italy vs. Wales), 26 June (Italy vs. Austria), 2 July (Belgium vs. Italy). Semifinal and final were held on 6 July (Italy vs. Spain, Italy won the penalty shoot-out 4-2) and 11 July (Italy vs. England, Italy won the penalty shoot-out 3-2). The effect of matches was analyzed on the day that the match was played and the day after to avoid misclassification, as some of the matches were played in the evening. The control period (20 days) consisted of the days before and after 2 days of Italian team matches.

The study protocol was approved by the ethics committee of the Magna Graecia University of Catanzaro, Italy and all data were blindly analyzed. All patients provided written informed consent. The study conforms to the principles outlined in the Declaration of Helsinki.

It was not appropriate or possible to involve patients or the public in the design, or conduct, or reporting, or dissemination plans of this research.

### Statistical analysis

The distribution of variables was evaluated using the Kolmogorov–Smirnov test. Continuous variables following a normal distribution were presented as mean ± SD and compared using the unpaired-sample Student's *t*-test. Otherwise, variables that didn't follow normal distribution were presented as median (interquartile range [IQR]) and were compared with the Mann–Whitney *U* test. Categorical data were presented as numbers and percentages and compared with the chi-square test or Fisher exact tests. A *p*-value of <0.05 was considered to indicate statistical significance; all tests were two-sided.

We used Poisson regression with a time effect of 0 and +1 day to assess the effect of matches (6).

We calculated incidence ratios for the days of matches played by the Italian team and the remaining days of matches not involving the Italian team as the control period.

Statistical analyses were performed using the MedCalc Statistical Software version 14.8.1 (MedCalc Software, Ostend, Belgium).

## Results

### Baseline characteristics

A total of 1,235 patients admitted to Cardiac Care Units (CCUs) during the whole period of the UEFA Euro 2020 Football Championship (between 11 June and 12 July 2021) were analyzed. Of these, 499 were admitted during days of matches involving Italy, and 736 were admitted during the remaining days.

Among the patients, there were no significant differences in term of: male gender (66.7 vs. 68.7%, *P* = 0.50), mean age (66.62 ±12.2 years vs. 67.52 ± 12.31, *P* = 0.44), arterial hypertension (72.3 vs. 70.1%, *P* = 0.44), family history of cardiovascular disease (26.1 vs. 70.1%, *P* = 0.93), dyslipidemia (56.9 vs. 58.3%, *P* = 0.66), smoking (39.5 vs. 40.2%, *P* = 0.66), diabetes (29.1 vs. 26.2%, *P* = 0.27) and previous acute myocardial infarction (17.6 vs. 18.1%, *P* = 0.88). All the other parameters did not differ between the two groups except for body mass index (BMI) which was greater in the patients hospitalized during the control period in comparison with those hospitalized during days of matches involving Italy (29 ± 3.9 vs. 31.2 ± 3.9, *p* < 0.0001) (Table 1).

**Table 1:** Baseline characteristics.

	**Patients hospitalized during days of matches involving Italy (*n =* 499)**	**Patients hospitalized during control period** **(*n =* 736)**	***p*-value**
Male sex (*n*, %)	333, (66.7%)	506, (68,7%)	0.50
Age (*y*, ±SD)	66.95 ± 13.31	67.52 ± 12.31	0.44
STEMI (*n*, %)	205, (41.1%)	287 (39.0%)	0.49
NSTEMI (*n*, %)	213 (42.6%)	343 (46.6%)	0.18
UA (*n*, %)	63 (12.6%)	93 (12.6%)	0.93
TTS (*n*, %)	18 (3.6)	13 (1.8%)	0.07
Weight (kg, ±SD)	78.39 ±15.91	77 ± 15.07	0.12
Height (cm, ±SD)	168.65 ±12.68	169.04 ± 10.06	0.13
BMI (Kg/m^2^, ±SD)	29 ± 3.9	31.2 ± 3.9	**<0.0001**
Hypertension (*n*, %)	361 (72.3%)	516 (70.1%)	0.44
Family history CVD (*n*, %)	130 (26.1%)	189 (25.7%)	0.93
Dyslipidemia (*n*, %)	284 (56.9%)	429 (58.3%)	0.66
Active smoker (*n*, %)	197 (39.5%)	296 (40.2%)	0.85
Diabetes (*n*, %)	145 (29.1%)	192 (26.1%)	0.27
Prior TIA/stroke (*n*, %)	21 (4.2%)	24 (3.3%)	0.50
Prior AMI (*n*, %)	88 (17.6%)	133 (18.1%)	0.88
Prior PCI (*n*, %)	100 (20.1%)	132 (17.9%)	0.36
Prior CABG (*n*, %)	17 (3.4%)	34 (4.6%)	0.37
CKD (*n*, %)	70 (14.1%)	96 (13.0%)	0.64
Atrial fibrillation (*n*, %)	40 (8.0%)	66 (9.0%)	0.61
COPD (*n*, %)	35 (7.0%)	45 (6.1%)	0.60
Hemoglobin (g/dl, ±SD)	13.47 ± 1.79	13.59 ± 1.91	0.27
Creatinine (mg/dl, ±SD)	1.07 ± 0.67	1.1 ± 0.80	0.49
eGFR (ml/min, ±SD)	82 ± 34.7	79 ± 34.54	0.13
LDL-Chol (mg/dl, ±SD)	113.13 ± 45.20	110.58 ± 41.09	0.30

STEMI, ST-elevation myocardial infarction; NSTEMI, No ST-elevation myocardial infarction; UA, unstable angina; TTS, Takotsubo; BMI, body mass index; CVD, cardiovasculardisease; TIA, transient ischemic attack; AMI, acute myocardial infarction. Bold values indicate statistically significant results.

### Incidence of ACE during the UEFA Euro 2020 football championship

On the days of all matches involving the Italian team, there was no difference in the incidence of ACS [IRR 0.98 (0.87–1.11; *P* = 0.80)]. In particular, the comparative analysis of this group showed no differences in the frequency of admissions due to STEMI [IRR 1.03(0.86–1.24), *P* = 0.72], NSTEMI [IRR 0.97(0.81–1.15), *P* = 0.71], unstable angina [IRR 0.90(0.64–1.25), *P* = 0.52], and TTS [IRR 1.66(0.80–3.4), *P* = 0.14] compared to the control period (Table 2A).

**Table 2A:** Incidence of ACE on days during matches involving Italy, as compared with days during the control period.

	**Incidence rate during days of matches involving Italy**	**Incidence rate during control period**	**Incidence rate difference**	**Incidence rate ratio**	***P***
STEMI	0.40 (0.35–0.46)	0.39 (0.35–0.44)	0.013 (−0.06–0.08)	1.03 (0.86–1.24)	0.72
NSTEMI	0.44 (0.38–0.50)	0.45 (0.40–0.50)	−0.014 (−0.09–0.06)	0.97 (0.81–1.15)	0.71
UA	0.12 (0.09–0.16)	0.14 (0.11–0.17)	−0.01 (−0.05–0.03)	0.90 (0.64–1.25)	0.52
TTS	0.04 (0.02–0.06)	0.02 (0.01–0.03)	0.015 (0.004–0.034)	1.66 (0.80–3.48)	0.14

STEMI, ST-elevation myocardial infarction; NSTEMI, No ST-elevation myocardial infarction; UA, unstable angina; TTS, Takotsubo.

**Figure 1:**
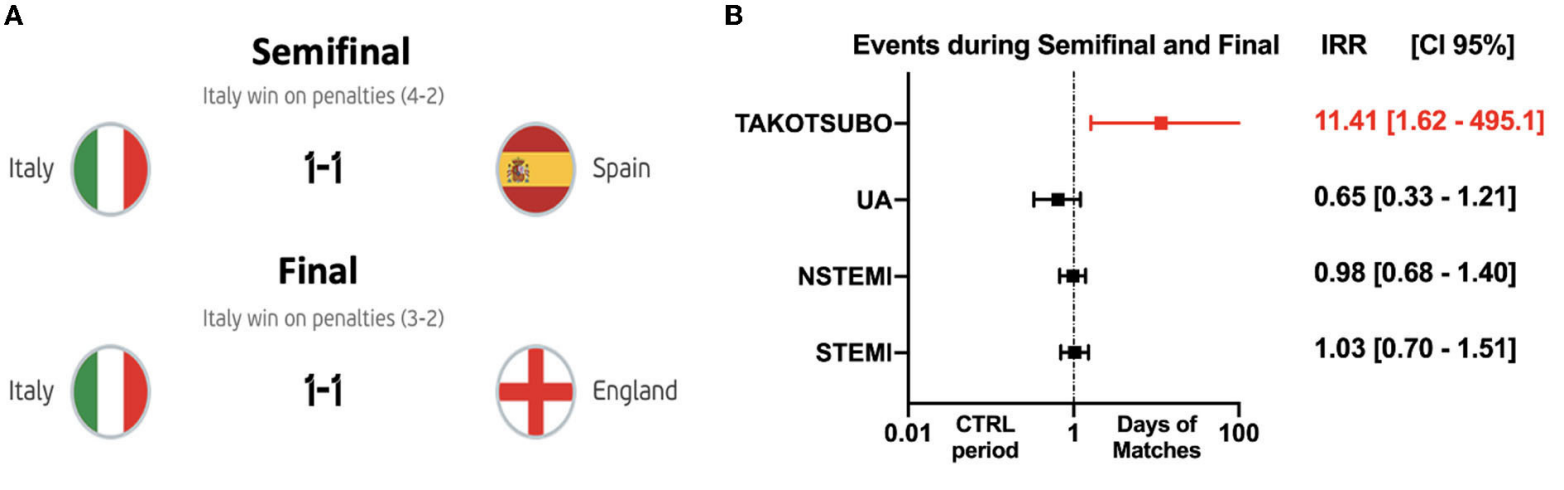
Incidence rate ratios for ACE on days during the semifinal and final of UEFA Euro 2020 Football Championship, as compared with days during the control period. ACE during the semifinal and final, the most stressful matches ended with penalties, and the victory of the Italian team against Spain and England **(A)**, showed a much more relevant increase in the incidence of Takotsubo Syndrome by a factor of 11.41 (*p* < 0.003) as compared with the control period. No differences were found in the incidence of ACS (*P* = 0.57) **(B)**.

Interestingly, ACE during the semifinal and final, the most stressful matches that ended with penalties and victory for the Italian team, showed a much more relevant increase in the incidence of Takotsubo Syndrome by a factor of 11.41 (1.6 – 495.1, *P* < 0.003) as compared with the control period (Figure 1). No differences were found in the incidence of ACS [IRR 0.93(0.74–1.18), *P* = 0.57] (Table 2B). In particular, we found 58 STEMI, 56 NSTEMI, 16 unstable angina, and 10 TTS (Table 4).

**Table 2B:** Incidence of ACE on semifinal and final matches, as compared with days during the control period.

	**Incidence rate during days of matches involving Italy**	**Incidence rate during control period**	**Incidence rate difference**	**Incidence rate ratio**	***P***
STEMI	0.39 (0.29–0.50)	0.38 (0.29–0.48)	0.011 (−0.13–0.15)	1.03(0.70–1.51)	0.88
NSTEMI	0.42 (0.32–0.54)	0.43 (0.34–0.55)	−0.009 (−0.16–0.14)	0.98 (0.68–1.40)	0.90
UA	0.12 (0.07–0.19)	0.18 (0.12–0.26)	−0.06 (−0.15–0.02)	0.65 (0.33–1.21)	0.15
TTS	0.07 (0.03–0.13)	0.01 (0.0002–0.03)	0.06 (0.02–0.11)	11.41 (1.62-495.1)	**0.0034**

STEMI, ST-elevation myocardial infarction; NSTEMI, No ST-elevation myocardial infarction; UA, unstable angina; TTS, Takotsubo. Bold values indicate statistically significant results.

### Characteristics of patients with Takotsubo Syndrome

Among patients with TTS on days when the Italian team played, most were female (70%) with a mean age of 60.7 ± 5.5 y.o. and BMI 24.3 ± 3.02. The proportion with hypertension was 60%, dyslipidemia (20%), and the 30% were active smokers. Nobody had diabetes or known coronary artery disease. Only one suffered from chronic kidney syndrome on dialysis (Table 3).

**Table 3:** Characteristics of patients with Takotsubo during semifinal and final matches.

**Age, *y***	**66**	**63**	**54**	**67**	**52**	**66**	**59**	**52**	**59**	**69**
**Gender**	**F**	**F**	**F**	**F**	**F**	**F**	**F**	**M**	**M**	**M**
**Weight, kg**	**70**	**65**	**58**	**52**	**60**	**80**	**65**	**104**	**70**	**65**
**Height, cm**	**178**	**165**	**165**	**160**	**165**	**165**	**162**	**175**	**175**	**168**
**BMI**	**22.1**	**23.9**	**21.3**	**20.3**	**22.0**	**29.4**	**24.8**	**34**	**22.9**	**23.0**
**Hypertension**	**+**	**–**	**+**	**–**	**+**	**+**	**–**	**+**	**–**	**+**
**Dyslipidemia**	**–**	**+**	**–**	**–**	**–**	**–**	**–**	**–**	**–**	**+**
**Active smoker**	**–**	**+**	**–**	**–**	**–**	**–**	**–**	**+**	**+**	**–**
**Diabetes**	**–**	**–**	**–**	**–**	**–**	**–**	**–**	**–**	**–**	**–**
**Previous stroke or TIA**	**–**	**–**	**–**	**–**	**–**	**–**	**–**	**–**	**–**	**–**
**Previous AMI**	**–**	**–**	**–**	**–**	**–**	**–**	**–**	**–**	**–**	**–**
**Previous PCI**	**–**	**–**	**–**	**–**	**–**	**–**	**–**	**–**	**–**	**–**
**Previous CABG**	**–**	**–**	**–**	**–**	**–**	**–**	**–**	**–**	**–**	**–**
**CKD**	**–**	**–**	**+**	**–**	**–**	**–**	**–**	**–**	**–**	**–**
**AF**	**–**	**–**	**–**	**–**	**–**	**–**	**–**	**–**	**–**	**–**
**COPD**	**–**	**–**	**–**	**–**	**–**	**–**	**–**	**–**	**–**	**–**
**Hemoglobin, g/dl**	**12**	**13.7**	**11.7**	**12**	**11.3**	**12.9**	**12.5**	**14.3**	**14.9**	**14.3**
**Creatinine, mg/dl**	**0.86**	**0.77**	**9.34**	**0.8**	**0.64**	**0.69**	**0.68**	**0.8**	**0.93**	**1.10**
**eGFR**	**71**	**77**	**6**	**56**	**97**	**101**	**91**	**169**	**85**	**58**
**LDL-Chol, mg/dl**	**70**	**–**	**160**	**126**	**135**	**109**	**113**	**161**	**168**	**163**
**Hs-Troponin ng/ml**	**719**	**4,015**	**909**	**2,786**	**1,186**	**175**		**964**	**350**	**50**
**LVEF, %**	**50**	**32**	**35**	**44**	**37**	**40**	**30**	**46**	**56**	**63**
**Apical ballooning**	**+**	**+**	**+**	**+**	**+**	**+**	**+**	**+**	**+**	**+**
**sPAP, mmHg**	**31**	**57**	**40**	**34**	**30**	**–**	**–**	**–**	**25**	**–**
**TAPSE, mm**	**19**	**19**	**–**	**20**	**20**	**20**	**–**	**20**	**22**	**–**

BMI, body mass index; CVD, cardiovascular disease; TIA, transient ischemic attack; AMI, acute myocardial infarction; LVEF, left ventricle ejection fraction; PAPs, pulmonary artery pressure; TAPSE, tricuspid annular plane excursion.

**Table 4:** ACE frequency in different periods.

	**Patients hospitalized during semifinal and final matches**	**Patients hospitalized during other days of matches involving Italy**	**Patients hospitalized during control period**
STEMI, n (mean/d)	58 (14.5)	147 (14.7)	287 (14.4)
NSTEMI, n (mean/d)	56 (14)	157 (15.7)	343 (17.92)
UA, n (mean/d)	16 (4)	47 (4.7)	93 (4.7)
TTS, n (mean/d)	10 (2.5)	8 (0.8)	13 (0.7)

STEMI, ST-elevation myocardial infarction; NSTEMI, No ST-elevation myocardial infarction; UA, unstable angina; TTS, Takotsubo.

**Figure 2:**
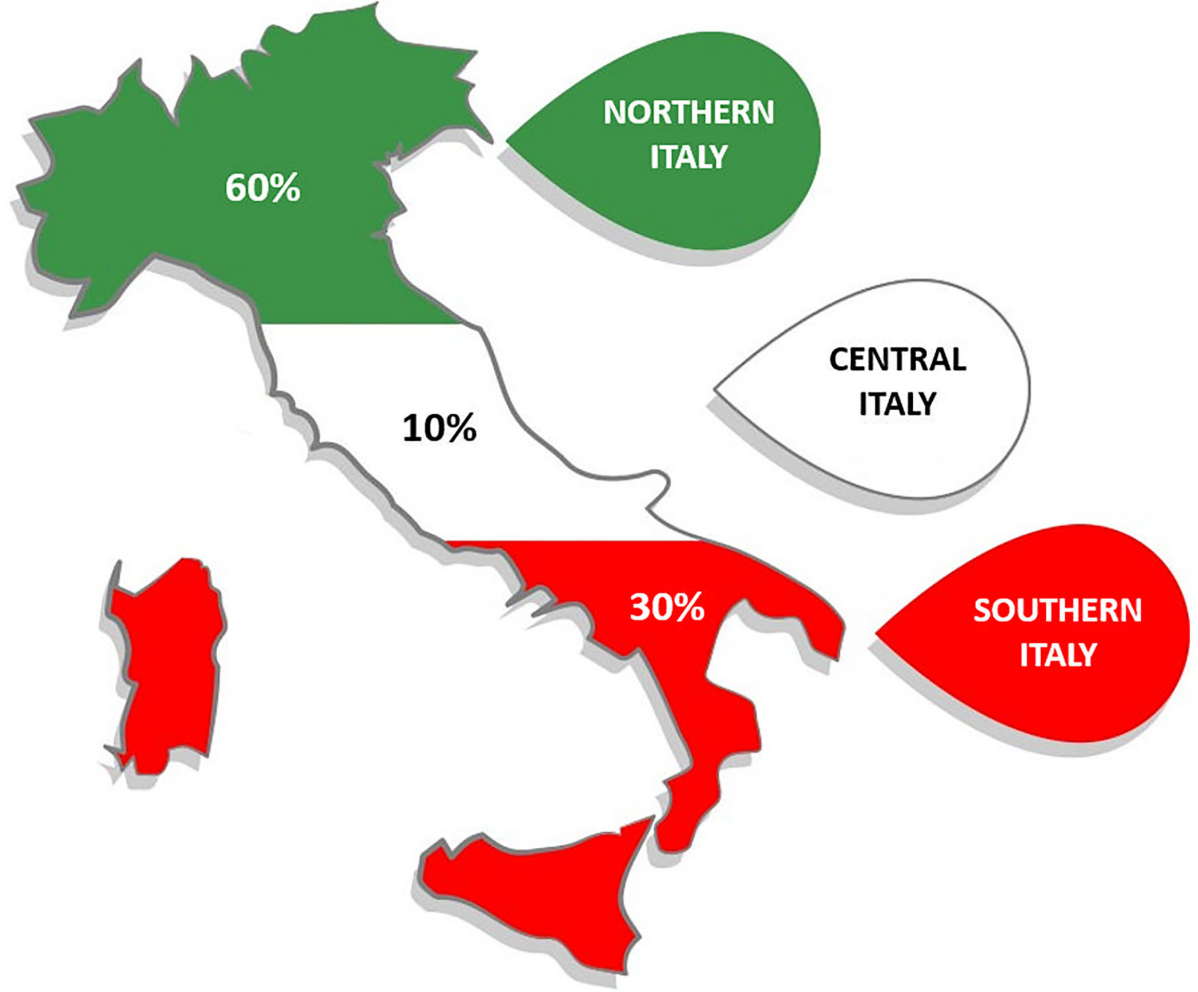
Geographic distribution of patients with Takotsubo Syndrome on days during the semifinal and final of UEFA Euro 2020 Football Championship.

Among patients with TTS, on days during the semifinal and final of the UEFA Euro 2020 Football Championship, 60% were admitted to centers in northern Italy, 10% to centers in central Italy, and 30% to centers in southern Italy (Figure 2).

## Discussion

The major finding of the present study was the significantly increased risk of Takotsubo syndrome induced by a positive event such as the semifinal and final of the UEFA Euro 2020 Football Championship, ended with the victory of the Italian team.

Since its description in 1990 (7), TTS has been defined as a syndrome precipitated by severe negative emotional stress such as pain, anger, or fear (8–10).

These precipitating causes have led to the popular definition of “broken heart syndrome” (11). It is also recognized that TTS predominantly affects postmenopausal women (12). Negative emotional stress can result in excessive or inappropriate stimulation of the sympathetic system or parasympathetic withdrawal (13).

However, TTS can be triggered by not only negative but also positive life events. Anecdotal reports (14–16) and data on the Takotsubo Registry reported that also positive emotions can trigger TTS, called the “Happy Heart Syndrome” (17).

In an international registry, the clinical presentation of patients with “happy heart syndrome” was similar to those with “broken heart syndrome” (17). Symptoms such as chest pain or electrocardiographic parameters, laboratory findings, and the 1-year outcome did not differ in the two forms (17).

The possible mechanisms of football matches induced TTS are not fully understood and might be related to an exaggerated sympathetic stimulation, especially during very stressful matches like finals. The role of the insula in the recognition of emotions such as anger and happiness through interoceptive processing has been previously demonstrated (18). Also, genetically driven variation in the response of brain regions underlying human emotional behavior may affect the differential excitability of the amygdala to emotional stimuli (19).

Early studies have shown an association between watching football matches and an increased risk of an ACS (1, 2).

Interestingly, our study did not find an increase in the rates of ACS on the days of football matches involving Italy during the entire competition or during final and semifinal matches.

However, these findings are consistent with most previous studies (3, 4, 20).

In particular, Barone-Adesi et al. (3) did not find an increase in the rates of admission for AMI on the days of football matches involving Italy during three international football competitions. In addition, in another study watching football matches was also not associated with an increase in cardiac events (4). Conversely, the study of Wilbert-Lampen et al. (1) showed an association between watching football matches and an increased risk of an ACS in the German population during the Football World Cup 2006. The authors reported a 2.7-fold increase in cardiac emergencies in the 12 h before and after football matches involving the German team (1). However, the results of this study were not confirmed by more extensive studies (3, 4, 20) and should not be generalized.

However, there are no systematic updated data available on the role of acute emotional stress induced by football championships as a trigger for Takotsubo Syndrome in the contemporary era.

We showed for the first time, in a large national registry, a significant increase in TTS incidence during stressful football matches associated with a positive event as the victory of the UEFA Euro 2020 Football Championship finals by the Italian team. Moreover, we found a TTS prevalence of 30% among male patients, while recent studies reported a prevalence of TTS approximately in 10% of the male sex (12). This data, although apparently surprising, can be explained by the fact that soccer is usually more followed by a male audience than a female one, especially in Italy as reported in a recent survey of 1,029 respondents^1^

Only a few reports described a TTS after an acute emotional stress event watching football matches and were all triggered by negative events (5, 21, 22). Fijalkowski et al. described a TTS on a male after an acute emotional stress event caused by the defeat of his favorite soccer team during the Euro 2012 cup (5). Elamin et al. presented a case of a football supporter of Sheffield United who was admitted to the hospital with chest pain following a last-minute goal by the opposing team (21). Y-Hassan et al. reported a case in which a missed penalty kick triggered coronary death in the husband and TTS in the wife (22).

To date, no systematic studies or registries have shown an association between TTS and important football championships after positive emotional stress.

## Limitations of the study

Our study has some limitations. First, we did not examine data on stroke, arrhythmias, and sudden death. However, sudden death is much less frequent than hospital admission for ACS.

Second, we assumed that patients with events on match days had indeed been watching the match. However, it is likely that the hospitalized patients have seen the matches considering the TV share over 21 million views during the finals in Italy.

Third, we do not have any information on the time of the onset of symptoms. However, by analyzing the effect of matches on the day of the match and the day after we avoided a misclassification, as some of the matches were watched in the evening and thus were miscounted on a control day.

Fourth, we have no data about recent (or concomitant) COVID-19 infection. Moreover, we have not performed a comparison with the previous year to avoid confounders related to the pandemic.

Fifth, we do not have any data on coronary angiography, CMR, IVUS or OCT findings and long-term follow-up.

Moreover, since the control period consisted of the days before and after the matches, it is unlikely that our results are due to the effects of unmeasured potential confounders such as temperature and pollution that were similar during the entire tournament.

## Conclusions

The data of this national Registry demonstrated an association between the semifinal and final of the UEFA Euro 2020 Football Championship and TTS suggesting that it can be triggered by also positive emotions. Conversely, no differences were found in the incidence of ACS. Further prospective studies should be planned to assess the relative association risk of football matches and TTS.

## Data availability statement

The raw data supporting the conclusions of this article will be made available by the authors, without undue reservation.

## Ethics statement

The studies involving human participants were reviewed and approved by the Ethics Committee of the Magna Graecia University of Catanzaro, Italy. The patients/participants provided their written informed consent to participate in this study.

## Author contributions

All authors listed have made a substantial, direct, and intellectual contribution to the work and approved it for publication.

## Conflict of interest

The authors declare that the research was conducted in the absence of any commercial or financial relationships that could be construed as a potential conflict of interest.

## Publisher's note

All claims expressed in this article are solely those of the authors and do not necessarily represent those of their affiliated organizations, or those of the publisher, the editors and the reviewers. Any product that may be evaluated in this article, or claim that may be made by its manufacturer, is not guaranteed or endorsed by the publisher.

## Footnotes

^1^Share of Italians interested in soccer in 2020. https://www.statista.com/statistics/1127483/share-of-italians-interested-in-football-by-gender/.

## References

[1] Wilbert-Lampen U Leistner D Greven S. Cardiovascular Events during World Cup Soccer. N Engl J Med. (2008) 358:475–83. 10.1056/NEJMoa0707427 18234752

[2] Carroll D. Admissions for myocardial infarction and World Cup football: database survey. BMJ. (2002) 325:1439–42. 10.1136/bmj.325.7378.1439 12493655 PMC139028

[3] Barone-Adesi F Vizzini L Merletti F Richiardi L. It is just a game: lack of association between watching football matches and the risk of acute cardiovascular events. Int J Epidemiol. (2010) 39:1006–13. 10.1093/ije/dyq007 20211850

[4] Niederseer D Thaler CW Egger A Niederseer MC Ploderl M Niebauer J. Watching soccer is not associated with an increase in cardiac events. Int J Cardiol. (2013) 170:189–94. 10.1016/j.ijcard.2013.10.066 24182671

[5] Fijalkowski M Fijalkowska M Nowak R Rynkiewicz A. Takotsubo cardiomyopathy in a male during a Euro 2012 football match. Clin Res Cardiol. (2013) 102:319–21. 10.1007/s00392-013-0536-7 23354629 PMC3601249

[6] Sahai H Khurshid A. Statistics in Epidemiology: Methods, Techniques, and Applications. (1996). Boca Raton, FL: CRC Press, Inc.

[7] Sato HTH Uchida T Dote K Ishihara M. Tako-tsubo-like left ventricular dysfunction due to multivessel coronary spasm. In: Kodama K Haze K Hori M. Clinical Aspect of Myocardial Injury: From Ischemia to Heart Failure (in Japanese). (1990) (Kagakuhyoronsha: Kagakuhyoronsha Publishing Co.), 56–64.

[8] Akashi YJ Goldstein DS Barbaro G Ueyama T. Takotsubo cardiomyopathy: a new form of acute, reversible heart failure. Circulation. (2008) 118:2754–62. 10.1161/CIRCULATIONAHA.108.767012 19106400 PMC4893309

[9] Sharkey SW Lesser JR Zenovich AG Maron MS Lindberg J Longe TF. Acute and reversible cardiomyopathy provoked by stress in women from the United States. Circulation. (2005) 111:472–479. 10.1161/01.CIR.0000153801.51470.EB 15687136

[10] Templin C Ghadri JR Diekmann J Napp LC Bataiosu DR Jaguszewski M. Clinical features and outcomes of takotsubo (stress) cardiomyopathy. N Engl J Med. (2015) 373:929–38 10.1056/NEJMoa1406761 26332547

[11] Brandspiegel HZ Marinchak RA Rials SJ Kowey PR. A broken heart. Circulation. (1998) 98:1349 10.1161/01.CIR.98.13.1349 9751687

[12] Pattisapu VK Hao H Liu Y Nguyen TT Hoang A Bairey Merz CN. Sex- and age-based temporal trends in Takotsubo syndrome incidence in the United States. J Am Heart Assoc. (2021) 10:e019583. 10.1161/JAHA.120.019583 34641717 PMC8751899

[13] Omerovic E Citro R Bossone E Bjorn Redfors Johannes Backs Bastian Bruns. Pathophysiology of Takotsubo Syndrome—a joint scientific statement from the Heart Failure Association Takotsubo Syndrome Study Group and Myocardial Function Working Group of the European Society of Cardiology - Part 1: Overview and the central role for catecholamines and sympathetic nervous system. Eur J Heart Fail. (2021). 10.1002/ejhf.2400 34907620

[14] Qin D Patel SM Champion HC. “Happiness” and stress cardiomyopathy (apical ballooning syndrome/takotsubo syndrome). Int J Cardiol. (2014) 172:e182–83. 10.1016/j.ijcard.2013.12.140 24507745

[15] Nagai M Kobayashi Y Kobatake H Dote K Kato M Oda N. Happy heart syndrome: a case of Takotsubo syndrome with left internal carotid artery occlusion. Clin Auton Res. (2020) 30:347–50. 10.1007/s10286-020-00696-z 32451755

[16] Allen D Parmar G Ravandi A Hussain F Kass M. Happiness can break your heart: a rare case of takotsubo cardiomyopathy after good news. Can J Cardiol. (2015) 31:228.e1–228.e2 10.1016/j.cjca.2014.11.011 25661563

[17] Ghadri JR Sarcon A Diekmann J Bataiosu DR Cammann VL Jurisic S. Happy heart syndrome: role of positive emotional stress in takotsubo syndrome. Eur Heart J. (2016) 37:2823–9. 10.1093/eurheartj/ehv757 26935270 PMC5841222

[18] Terasawa Y Motomura K Natsume A Iijima K Chalise L Sugiura J. Effects of insular resection on interactions between cardiac interception and emotion recognition. Cortex. (2021) 137:271–81. 10.1016/j.cortex.2021.01.011 33662691

[19] Hariri AR Mattay VS Tessitore A Kolachana B Fera F Goldman D. Serotonin transporter genetic variation and the response of the human amygdala. Science. (2002) 297:400–3. 10.1126/science.1071829 12130784

[20] Toubiana L Hanslik T Letrilliart L. French cardiovascular mortality did not increase during 1996 European football championship. BMJ. (2001) 322:1306. 10.1136/bmj.322.7297.1306 11403063 PMC1120392

[21] Elamin N Hashmi I Tilney M Grech E. When Blades broke my heart. Br J Cardiol. (2021) 28:19. 10.5837/bjc.2021.019 35747457 PMC8988219

[22] Y-Hassan S Feldt K Stålberg M. A missed penalty kick triggered coronary death in the husband and broken heart syndrome in the wife. Am J Cardiol. (2015) 116:1639–42. 10.1016/j.amjcard.2015.08.033 26410607

