# Supplementary material for: The impact of UEFA Euro 2020 football championship on Takotsubo Syndrome: Results of a multicenter national registry
Source: Front Cardiovasc Med. 2022 Sep 28;9:951882. doi: 10.3389/fcvm.2022.951882 (PMC9554214; doi:10.3389/fcvm.2022.951882)
Supplement: Supplementary file 1 [file Data_Sheet_1.PDF]

Supplementary Table 1 – List of enrolling centers

Italian Centers	Number of patients enrolled n (%)
OSPEDALE SPOKE PAOLA- CETRARO PO PAOLA - UTIC CARDIOLOGIA PAOLA – CS	**16 (1.21)**
ISTITUTO N. ROSANO CLINICA TRICARICO - DIVISIONE CARDIOVASCOLARE BELVEDERE MARITTIMO – CS	**17 (1.28)**
OSPEDALE CIVILE PUGLIESE - UTIC-EMODINAMICA E CARD. INTERVENTISTICA CATANZARO – CZ	**53 (3.99)**
OSPEDALE GIOVANNI PAOLO II - U.O. CARDIOLOGIA E UTIC LAMEZIA TERME – CZ	**11 (0.82)**
OSPEDALE SANTA MARIA DEGLI UNGHERESI - UOC CARDIOLOGIA-UTIC POLISTENA – RC	**11 (0.82)**
AZ. OSPEDALIERA S. ANNA E S. SEBASTIANO - U.O. CARDIOLOGIA-UTIC CASERTA – CE	**29 (2.19)**
FONDAZIONE EVANGELICA BETANIA - UOS CARDIOLOGIA NAPOLI – NA	**8 (0.60)**
OSPEDALE SANTA MARIA DELLE GRAZIE - U.O. CARDIOLOGIA – UTIC POZZUOLI – NA	**45 (3.39)**
AOU S. GIOVANNI DI DIO-RUGGI D'ARAGONA - U.O.C. UTIC SALERNO – SA	**57 (4.30)**
PRESIDIO OSPED. S. MARIA DELLA SPERANZA - U.O. DI CARDIOLOGIA BATTIPAGLIA – SA	**2 (0.15)**
OSPEDALE DI BENTIVOGLIO - U.O. DI CARDIOLOGIA BENTIVOGLIO - BO	**11 (0.82)**
OSPEDALE POLICLINICO - UO CARDIOLOGIA MODENA – MO	**20 (1.51)**
PO SANTA MARIA NUOVA - AUSL RE IRCCS - SOC CARDIOLOGIA OSPEDALIERA REGGIO EMILIA – RE	**48 (3.62)**
ASUGI - POLO CARDIOLOGICO CATTINARA - S.O.C. CARDIOLOGIA TRIESTE – TS	**48 (3.62)**
POU SANTA MARIA DELLA MISERICORDIA - S.O.C. CARDIOLOGIA UDINE – UD	**50 (3.77)**
OSPEDALE FABRIZIO SPAZIANI - CARDIOLOGIA-UTIC FROSINONE – FR	**52 (3.92)**

OSPEDALE S. MARIA GORETTI - UOC MALATTIE CARDIOVASCOLARI LATINA – LT	**14 (1.06)**
POLICLINICO UMBERTO PRIMO - MALATTIE CARDIOVASCOLARI ROMA – RM	**19 (1.43)**
EUROPEAN HOSPITAL - U.O. CARDIOLOGIA INTERVENTISTICA ROMA – RM e AURELIA HOSPITAL - U.O.C CARDIOLOGIA ROMA – RM	**34 (2.56)**
OSPEDALE SANT'ANDREA DI ROMA - U.O.C. CARDIOLOGIA ROMA - RM	**24 (1.81)**
POLICLINICO TOR VERGATA - U.O. CARDIOLOGIA-CARD. INTERVENTISTICA ROMA – RM	**57 (4.29)**
ASST SPEDALI CIVILI – CARDIOLOGIA BRESCIA – BS	**31 (2.34)**
ASST SANTI PAOLO E CARLO - PO SAN PAOLO - UO CARDIOLOGIA E UTIC MILANO – MI	**22 (1.66)**
CLINICA SAN CARLO - U.O. CARDIOLOGIA-UNITA'CURE CORONARICHE PADERNO DUGNANO – MI	**21 (1.58)**
PRESIDIO OSPEDALIERO DI SARONNO - U.O.C. DI CARDIOLOGIA SARONNO – VA	**10 (0.75)**
ISTITUTO SCIENTIFICO INRCA - UO CARDIOLOGIA/UTIC/TELECARDIOLOGIA ANCONA – AN	**8 (0.60)**
OSPEDALE A. CARDARELLI - U.O. CARDIOLOGIA E UTIC CAMPOBASSO - CB	**16 (1.21)**
OSPEDALE GENERALE REGIONALE - DIVISIONE DI CARDIOLOGIA BOLZANO – BZ	**47 (3.54)**
OSPEDALE REGINA MONTIS REGALIS - U.O. CARDIOLOGIA-UTIC MONDOVI' – CN	**15 (1.13)**
AOU MAGGIORE DELLA CARITA' - CARDIOLOGIA II NOVARA – NO	**30 (2.26)**
OSPEDALE MAURIZIANO UMBERTO I - SC CARDIOLOGIA TORINO – TO	**32 (2.41)**
OSPEDALE DEGLI INFERMI - SC CARDIOLOGIA RIVOLI – TO	**51 (3.84)**
OSPEDALE UNICO DOMODOSSOLA-VERBANIA - SOC CARDIOLOGIA DOMODOSSOLA e OSPEDALI RIUNITI DOMODOSSOLA-VERBANIA - SOC CARDIOLOGIA – VB	**32 (2.41)**

OSPEDALE POLICLINICO - U.O. CARDIOLOGIA UNIVERSITARIA BARI - BA	**7 (0.53)**
OSPEDALE SAN PAOLO - CARDIOLOGIA-UTIC BARI – BA	**23 (1.73)**
AZIENDA OSP. CARDINALE G. PANICO - U.O. CARDIOLOGIA – UTIC TRICASE – LE	**32 (2.41)**
AZIENDA OSPEDALIERA G. BROTZU - STRUTTURA COMPLESSA DI CARDIOLOGIA - CAGLIARI – CA	**33 (2.49)**
A.O.U. CAGLIARI POLICLINICO MONSERRATO - SC CARDIOLOGIA UTIC MONSERRATO – CA	**41 (3.09)**
AZIENDA OSPEDALIERA CANNIZZARO - UOC CARDIOLOGIA CON UTIC ED EMODINAMICA CATANIA – CT	**33 (2.49)**
POLICLINICO P. GIACCONE - CARDIOLOGIA II CON EMODINAMICA PALERMO – PA	**13 (0.98)**
OSPEDALE E. MUSCATELLO - U.O. DI CARDIOLOGIA – UTIC AUGUSTA – SR	**2 (0.15)**
AOU CAREGGI - CARDIOLOGIA INTERVENTISTICA FIRENZE – FI	**21 (1.58)**
OSPEDALE APUANE - U.O. CARDIOLOGIA-UTIC MASSA – MS	**13 (0.98)**
AZIENDA OSPEDALIERA UNIVERSITARIA PISANA - U.O. MALATTIE CARDIOVASCOLARI – CISANELLO PISA – PI	**32 (2.41)**
PRESIDIO OSPEDALIERO CITTA' DI CASTELLO - U.O. DI CARDIOLOGIA CITTA' DI CASTELLO – PG	**12 (0.90)**
OSPEDALE GUBBIO-GUALDO TADINO - U.O. DI UTIC E CARDIOLOGIA - PG	**13 (0.98)**
AZIENDA OSPEDALIERA SANTA MARIA - S.C. DI CARDIOLOGIA TERNI – TR	**37 (2.79)**
AZIENDA OSPEDALIERA PADOVA - UOSD TERAPIA INTENSIVA CARDIOLOGICA PADOVA – PD	**41 (3.09)**
OSPEDALE CA' FONCELLO - U.O.C. CARDIOLOGIA TREVISO – TV	**32 (2.41)**
